# Oxidative Stress and Antioxidants in Aging

**DOI:** 10.3390/antiox13111288

**Published:** 2024-10-24

**Authors:** Silvia Dossena, Angela Marino

**Affiliations:** 1 Institute of Pharmacology and Toxicology, Paracelsus Medical University, 5020 Salzburg, Austria; 2 Research and Innovation Center Regenerative Medicine & Novel Therapies (FIZ RM&NT), Paracelsus Medical University, 5020 Salzburg, Austria; 3 Department of Chemical, Biological, Pharmaceutical and Environmental Sciences, University of Messina, 98166 Messina, Italy

Global aging represents a challenge for social health. The increase in life expectancy over the last century and the consequent aging of the general population impose the need to counteract age-related diseases and ensure healthy aging [1]. The aging process is tightly connected to oxidative stress-related perturbations of organ, tissue, and cell function [2]. On this basis, the Special Issue “Oxidative Stress and Antioxidants in Aging” was intended to collect the most recent research on the mechanisms underlying the impact of oxidative stress during aging on cells and cellular components, including DNA, organelles, membrane ion transport systems, and the cell redox system. In addition, the beneficial effects of antioxidants and possible mechanisms of cell adaptation to age-related conditions were also considered.

This Special Issue collected eight scientific papers, including six original articles and two reviews from outstanding researchers. Here, we offer a brief overview of these contributions, which can be divided into two main thematic areas. In particular, four manuscripts propose new insights into the physiological development and molecular mechanisms of aging and age-related conditions [3,4,5,6] and four manuscripts [7,8,9,10] report on the potential beneficial effect of natural antioxidants on age-related pathophysiological changes.

Regarding the contributions dealing with the mechanisms of aging and age-related conditions, Kim and co-authors examined the potential roles of superoxide dismutase (SOD) family members in skin aging [3]. SOD are highly compartmentalized enzymes that convert the superoxide anion (O2•−) to hydrogen peroxide (H_2_O_2_) and oxygen [11]. The authors found that the expression levels of SOD3, the extracellular matrix SOD isoform, were significantly reduced in the skin of old mice and humans compared to young counterparts, while SOD1 and SOD2 expression levels remained unchanged. The incubation of human foreskin fibroblasts with purified recombinant human SOD3 significantly reduced intracellular reactive oxygen species (ROS) and secretion of matrix metalloproteinase (MMP)-1, while increasing type I collagen secretion. These effects were particularly evident after exposure of cells to TNF-alpha, which greatly elevated intracellular ROS, and arose from gene transcriptional modulation. The authors also dissected the intracellular signaling pathway underlying the downregulation of MPP-1 and found NF-κB, ERK, and p38 MAPK to be involved. The observation of the increased production and reduced fragmentation of type I collagen was recapitulated in 3D cultures of foreskin fibroblasts. These findings point to SOD3 as a major player in preserving the integrity of the extracellular matrix of the skin during aging [3].

It is known that, during aging, a pro-inflammatory state is observed in most tissues, including the brain, which promotes the progression of major neurodegenerative diseases such as Parkinson’s disease (PD) [12,13]. Dysregulation of the local pro-oxidative and pro-inflammatory renin-angiotensin system (RAS) has been observed in aged brains and animal models of neurodegenerative diseases, including PD [14]. In this special issue, Quijano et al. explored whether brain RAS can activate the leucine-rich-repeat- and pyrin-domain-containing 3 (NLRP3) inflammasome during aging and in models of PD. The authors found that components of NLRP3 inflammasome were upregulated in the *substantia nigra* of aging rats, rat models of dopaminergic neurodegeneration, or following intraventricular injection of angiotensin II (AngII). These effects were counteracted by administering the angiotensin type I receptor (AT1) antagonist candesartan or AT1 receptor ablation. These findings identify the AngII/AT1 axis of the brain RAS as a druggable target in inflammaging and PD [4].

Exposure to drugs, and especially cancer chemotherapeutics, is a major source of oxidative stress, which might determine important side effects. Acute kidney injury (AKI) is the main dose-limiting factor of cisplatin treatment, and oxidative stress plays a major role in this context [15]. Older age represents one of the main risk factors for cisplatin nephrotoxicity [16]. Bushau-Sprinkle and collaborators formerly demonstrated that Na/H exchange regulatory factor isoform 1 (NHERF1) knock-out mice exhibit exacerbated cisplatin nephrotoxicity [17]. Now, these authors show that the kidneys of NHERF1 knock-out mice have a lower ratio of reduced to oxidized glutathione (GSH/GSSG) compared to the wild-type mice, revealing an underlying oxidative stress status. Genes involved in the glutathione synthesis, including the cystine/glutamate antiporter (*Slc7a11*) and the glutamate-cysteine ligase catalytic subunit (*Gclc*) were greatly upregulated in NHERF1-deficient kidneys after cisplatin exposure but the activity of γ-glutamyl transferase (GGT), which controls the intracellular recycling of Cys for the synthesis of GSH, failed to increase. These findings reveal a novel function of the scaffolding protein NHERF1 in reacting to an oxidative insult and protecting the kidney from cisplatin-induced AKI [5].

Aging carries a significant risk for many diseases, including cardiovascular disease, which represents the leading cause of death in developed countries [18]. In their review, Gu and Dayal focus on platelets as fundamental players in thrombotic vascular disease and oxidative stress targets. Platelets can produce ROS, which may act as important signaling molecules but can also lead to platelet hyperreactivity and increased risk of cardiovascular events. Several human and animal studies, including studies from these same authors, showed that glutathione peroxidase (GPx) plays a protective role in this context. Enhanced platelet reactivity is also observed in chronic low-grade inflammation associated with overproduction of the pro-inflammatory cytokine TNF-α, hyperlipidemia and atherosclerosis, type 2 diabetes mellitus, cancer, smoking, and COVID-19 infection, and oxidative stress is involved in each of these clinical settings. The authors highlight that it will be important to evaluate the effects of mitochondrial ROS and antioxidant enzymes in models of age-associated vascular disease and thrombosis in future studies [6].

Regarding the beneficial effect of antioxidants during aging, two manuscripts in this Special Issue utilized *Caenorhabditis elegans* as a model [7,8]. Ding and Zhao explored how astaxanthin, which was shown to reduce oxidative stress and extend the lifespan in *C. elegans* [19,20], can affect gene transcription [7]. Astaxanthin is a red carotenoid with strong antioxidant properties [21]. RNA sequencing was employed to identify the genes differentially expressed in *C. elegans* following exposure to astaxanthin for six days. A total of 190 mRNAs and six microRNAs (miRNAs) were found to be differentially expressed. Pathway analysis revealed that longevity-regulating pathways were significantly enriched, and the three most altered gene ontology (GO) terms were metabolic process, innate immune response, and lipid metabolic process. The target genes of differentially expressed miRNAs belonged to redox processes, determination of lifespan, and unfolded protein response. Analysis of protein-protein interaction networks identified central node genes functioning in fatty acid metabolism and lysosomal acidification. Importantly, these findings were corroborated in vivo by the evidence that exposure to astaxanthin limited fat accumulation in *C. elegans* [7]. The study indicates that, among other factors, down-regulation of genes involved in fat accumulation may promote lifespan extension.

Park and Park explored the beneficial effect of phlorizin in aging models established in *C. elegans* [8]. Phlorizin is a polyphenolic compound particularly abundant in the *Malus* genus. Its well-known ability to produce glycosuria and reduced absorption of glucose from the gastrointestinal tract stem from the inhibition of the sodium-glucose cotransporter in the epithelial cells of the renal proximal tubule and small intestine [22]. The authors show that dietary supplementation with phlorizin increased the survival of worms exposed to oxidative stress and UV irradiation. In addition, phlorizin significantly extended lifespan, delayed age-related decline of motility, reduced amyloid-beta toxicity and mortality induced by a high-glucose diet, and reverted degeneration of dopaminergic neurons in a PD model in *C. elegans*. These effects were linked to the upregulation of longevity, antioxidant, and autophagy genes [8]. These findings support further studies in mammals to assess the potential use of phlorizin as a novel anti-aging nutraceutical.

The next two contributions to this Special Issue explored the potential benefit of antioxidant nutraceuticals in age-related sensory impairment [9,10]. Age-related hearing loss (ARHL) and vision impairment are common conditions with limited therapeutic options that substantially affect the quality of life and lead to communication problems, decline in physical activity, and social isolation, which in turn increase the risk of depression and dementia in the elderly [23]. Del Mar Rivas-Chacón and collaborators explored the potential of cocoa flavonoids in age-related hearing loss by using three distinct inner ear cell lines exposed to oxidative stress. The cocoa extract protected the viability of auditory cells following exposure to H_2_O_2_, decreased the levels of reactive oxygen and nitrogen species, prevented oxidative DNA damage, increased the activity of the antioxidant enzymes SOD, catalase (CAT), and GPx, increased the total cell antioxidant capacity, ameliorated the metabolic status, and prevented apoptosis by inhibition of mitochondrial cell death pathways [9]. These findings, together with previous findings of the same research group showing improvements in hearing function, inhibition of the activation of age-related apoptotic signaling, and decreased oxidative stress in the rat cochlea [24,25,26], highlight the benefits of a polyphenol-enriched diet in the prevention of ARHL.

Age-related macular degeneration (ARMD) is an eye disease affecting the macula, causing loss of central vision, and is the leading cause of severe vision loss in adults over age 50. Oxidative damage in the retina, contributing to inflammation and angiogenesis, plays a fundamental role in this context [27]. In their review, Kushwah and collaborators summarized a substantial body of evidence on genetic and chemically induced animal and cellular models of wet and dry ARDM, all highlighting the central role of oxidative stress in this setting. Several clinical trials and preclinical studies showed a protective effect of antioxidants in ARMD, including vitamins C and E, β-carotene, zinc, lutein, zeaxanthin, ω-3 long-chain polyunsaturated fatty acids, resveratrol, glutathione, and N-acetyl-cysteine. Based on these promising results, the authors suggest that further work is warranted to establish the detailed mechanisms of action, bioavailability, and pharmacokinetics of antioxidants, including phytochemicals, to support their use in ARMD [10].

To conclude, this Special Issue highlights the fundamental role of oxidative stress in aging and age-related conditions and diseases, including skin aging, PD, cardiovascular disease, cancer, and sensory impairments. Solid evidence exists that antioxidants, including natural polyphenols, have the potential to attenuate, prevent, or delay age-related perturbations of normal physiological function. However, the translation of this concept to clinical practice mandates the identification of the targets of oxidative stress on a molecular and cellular level, as well as the precise understanding of the pharmacodynamics and pharmacokinetics of antioxidants in this context.
